# Simultaneous measurement of blood-flow velocity and regional wall motion with phase unwrapping

**DOI:** 10.1186/1532-429X-14-S1-P241

**Published:** 2012-02-01

**Authors:** Junmin Liu, James A White, Maria Drangova

**Affiliations:** 1 Imaging Research Laboratories, Robarts Research Institute, Schulich School of Medicine & Dentistry, The University of Western Ontario, London, ON, Canada; 2 Division of Cardiology, Department of Medicine, Schulich School of Medicine & Dentistry, The University of Western Ontario, London, ON, Canada; 3 Department of Medical Biophysics, Schulich School of Medicine & Dentistry, The University of Western Ontario, London, ON, Canada

## Summary

In this study we offer a unique approach to achieve simultaneous evaluation of tissue and blood flow velocities from a single “low-VENC” image dataset (i.e.: prescribed for measurement of tissue velocities). A novel phase unwrapping algorithm is tested for this purpose.

## Background

Doppler echocardiography-based techniques are routinely used to assess the heart and ventricular function by measuring blood flow and cardiac tissue velocity profiles. While these measures may similarly be obtained by phase contrast MRI, each require separate acquisitions and subsequent analysis to accommodate for vastly different velocities and therefore velocity encoding (VENC) values.

## Methods

MR imaging was performed on a 3.0-T whole-body scanner (MR 750, GE Medical Systems). Phase-contrast images with through-plane velocity-encoding were acquired in the short-axis plane with a retrospectively triggered 2D fast cine phase contrast pulse sequence (TR/TE, 7.3/4.4 ms; flip angle, 15 degree, slice thickness 8 mm) with first-order flow compensation in all dimensions to minimize artifacts from flow and motion. Three VENCs - 75, 20 and 10 cm/s - were used and the images acquired with VENC = 75 cm/s were used as a reference. The acquisition time (per VENC) was about 15 seconds, enabling acquisition within a single breath-hold. Thirty images were reconstructed per cardiac cycle. Phase unwrapping of the velocity data was achieved using an algorithm developed in our lab, which uses an orthogonal recursive approach to remove streaks that result following conventional 2D phase unwrapping.

## Results

**Figure 1:**
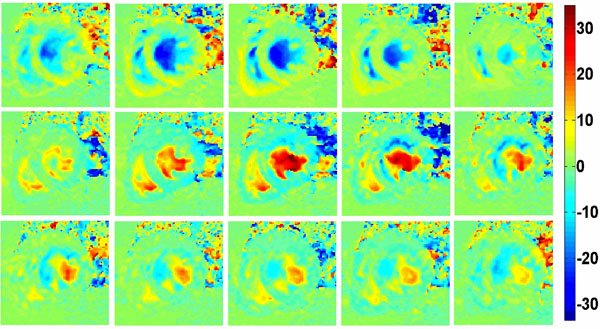
The odd-numbered time frames of the 30 unwrapped mid-ventricular phase images (VENC = 20 cm/s). The phase images have been corrected for background phase and are colour-coded in cm/s according to the scale on the right.

**Figure 2:**
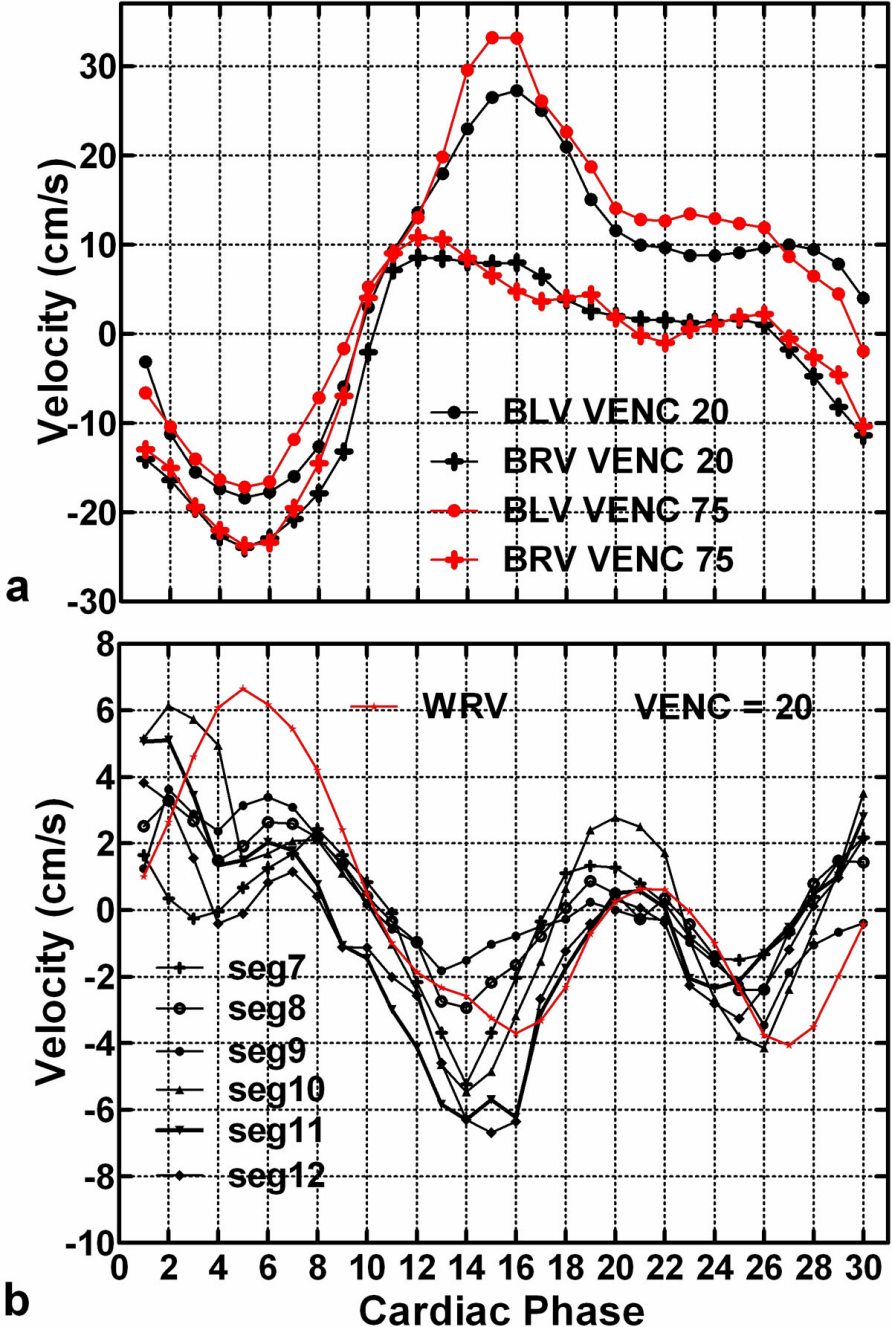
(a) The blood flow velocity in the LV (BLV) and RV (BRV). (b) Plots of regional wall motion of the middle ventricular plane. WRV denotes the lateral wall of the RV.

The unwrapped aliasing-free phase-contrast images (VENC = 20 cm/s) are shown in Fig. 1. The algorithm was insufficient to resolve phase wrapping produced form 10 cm/s acquired datasets, specifically during the early filling stage (detail not presented). The profiles of blood-flow in the LV and RV (VENC = 20cm/s) are close to that acquired with 75 cm/s (Fig. 2a). The results of mid-ventricula regional wall motion (Fig 2b) are similar to those reported by other groups. Additionally, we include the results of RV wall motion.

## Conclusions

Our preliminary results suggest that phase unwrapping using an orthogonal recursive technique can resolve phase wrap artifact from datasets acquired at VENC settings as low as 20 cm/s. This approach allows for simultaneous quantification of tissue and blood flow velocities within a single image acquisition and may be of value for the rapid assessment of diastolic dysfunction and left atrial pressures.

## Funding

Partial Ontario Research Fund and NSERC.

